# A Practical Treatise on the Diseases Peculiar to Woman, Illustrated by Cases Derived from Hospital and Private Practice

**Published:** 1855-07

**Authors:** 


A Practical Treatise on the Diseases peculiar to Woman, illustrated by cases derived from Hospital and Private Practice. By Samuel Ashwell, M. D., etc. Third American from the third and revised London edition. Philadelphia: Blanchard & Lea. 1855.

The work of Dr. Ashwell has been in the hands of the American public for some years, and it is unnecessary for us to do more than to announce its re-issue from the press of Messrs. Blanchard & Lea. We consider it one of the best works on that subject, and so we presume do our readers, at least that portion of them to whom its pages are familiar. To those unacquainted with it, our advice is, buy it and READ it. J.

